# Shared miRNA landscapes of COVID-19 and neurodegeneration confirm neuroinflammation as an important overlapping feature

**DOI:** 10.3389/fnmol.2023.1123955

**Published:** 2023-03-17

**Authors:** Sara Redenšek Trampuž, David Vogrinc, Katja Goričar, Vita Dolžan

**Affiliations:** Pharmacogenetics Laboratory, Institute of Biochemistry and Molecular Genetics, Faculty of Medicine, University of Ljubljana, Ljubljana, Slovenia

**Keywords:** COVID-19, neurodegeneration, neuroinflammation, Alzheimer’s disease, Parkinson’s disease, miRNA, biomarker

## Abstract

**Introduction:**

Development and worsening of most common neurodegenerative diseases, such as Alzheimer’s disease, Parkinson’s disease, and multiple sclerosis, have been associated with COVID-19 However, the mechanisms associated with neurological symptoms in COVID-19 patients and neurodegenerative sequelae are not clear. The interplay between gene expression and metabolite production in CNS is driven by miRNAs. These small non-coding molecules are dysregulated in most common neurodegenerative diseases and COVID-19.

**Methods:**

We have performed a thorough literature screening and database mining to search for shared miRNA landscapes of SARS-CoV-2 infection and neurodegeneration. Differentially expressed miRNAs in COVID-19 patients were searched using PubMed, while differentially expressed miRNAs in patients with five most common neurodegenerative diseases (Alzheimer’s disease, Parkinson’s disease, Huntington’s disease, amyotrophic lateral sclerosis, and multiple sclerosis) were searched using the Human microRNA Disease Database. Target genes of the overlapping miRNAs, identified with the miRTarBase, were used for the pathway enrichment analysis performed with Kyoto Encyclopedia of Genes and Genomes and Reactome.

**Results:**

In total, 98 common miRNAs were found. Additionally, two of them (hsa-miR-34a and hsa-miR-132) were highlighted as promising biomarkers of neurodegeneration, as they are dysregulated in all five most common neurodegenerative diseases and COVID-19. Additionally, hsa-miR-155 was upregulated in four COVID-19 studies and found to be dysregulated in neurodegeneration processes as well. Screening for miRNA targets identified 746 unique genes with strong evidence for interaction. Target enrichment analysis highlighted most significant KEGG and Reactome pathways being involved in signaling, cancer, transcription and infection. However, the more specific identified pathways confirmed neuroinflammation as being the most important shared feature.

**Discussion:**

Our pathway based approach has identified overlapping miRNAs in COVID-19 and neurodegenerative diseases that may have a valuable potential for neurodegeneration prediction in COVID-19 patients. Additionally, identified miRNAs can be further explored as potential drug targets or agents to modify signaling in shared pathways.

**Graphical Abstract:**
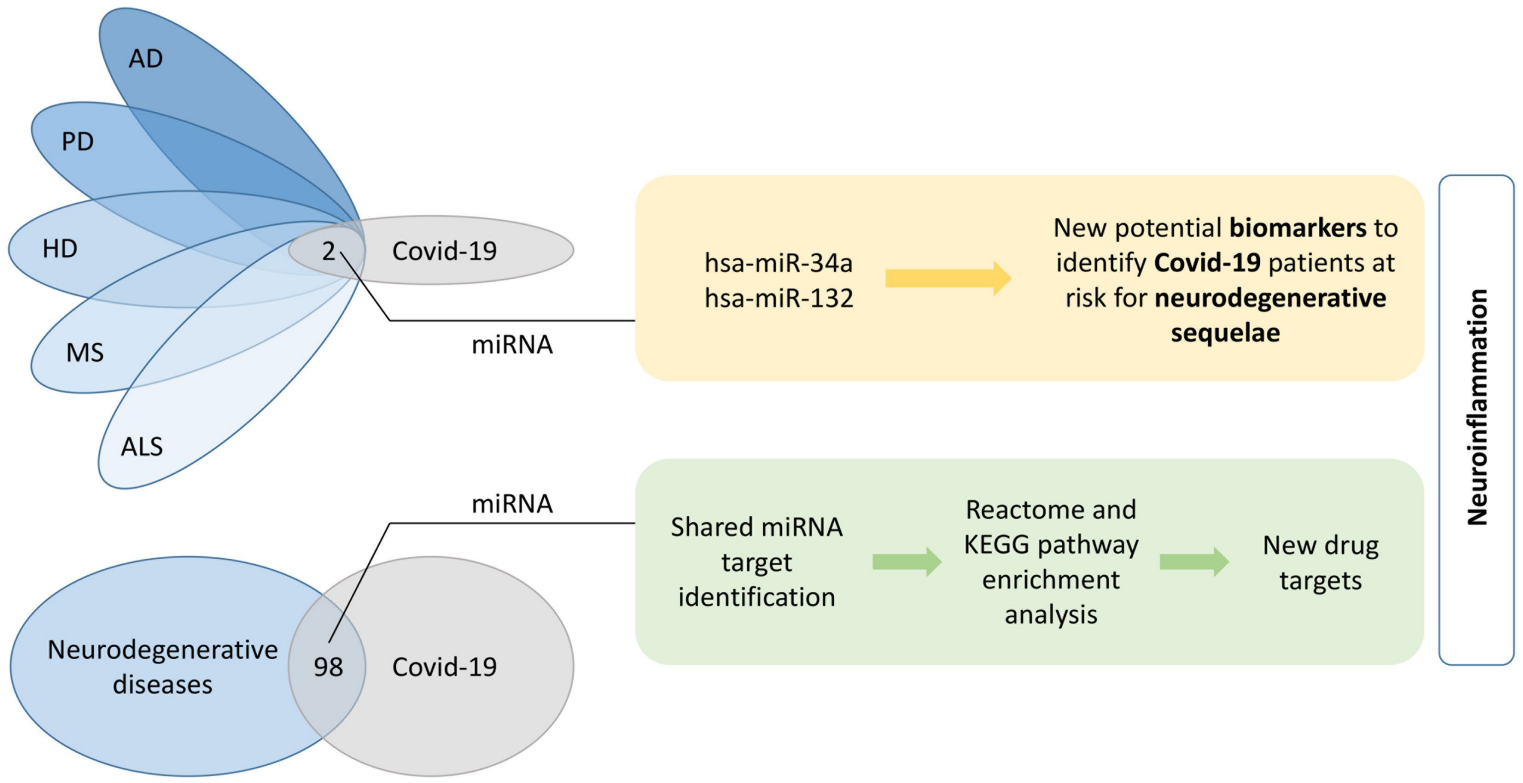
Shared miRNA molecules among the five investigated neurodegenerative diseases and COVID-19 were identified. The two overlapping miRNAs, hsa-miR-34a and has-miR-132, present potential biomarkers of neurodegenerative sequelae after COVID-19. Furthermore, 98 common miRNAs between all five neurodegenerative diseases together and COVID-19 were identified. A KEGG and Reactome pathway enrichment analyses was performed on the list of shared miRNA target genes and finally top 20 pathways were evaluated for their potential for identification of new drug targets. A common feature of identified overlapping miRNAs and pathways is neuroinflammation. AD, Alzheimer’s disease; ALS, amyotrophic lateral sclerosis; COVID-19, coronavirus disease 2019; HD, Huntington’s disease; KEGG, Kyoto Encyclopedia of Genes and Genomes; MS, multiple sclerosis; PD, Parkinson’s disease.

## 1. Introduction

Increasing evidence suggests that neurological deficits may develop due to infection with SARS-CoV-2 in a substantial proportion of patients (Heneka et al., 2020). These deficits may manifest acutely and sub-acutely, as well as within the long COVID-19 pathology, and are often referred to as neuro-COVID-19 (Chiappelli, 2020). About 36% of cases develop neurological symptoms, such as headache, nausea, anosmia, ageusia, myalgia/fatigue, confusion, disorientation, and vomiting (Jarrahi et al., 2020), of which 25% can be attributed to the direct involvement of the central nervous system (CNS; Mao et al., 2020). SARS-CoV-2 infection causes neurodegeneration (De Felice et al., 2020; Hascup and Hascup, 2020; Lippi et al., 2020; Karuppan et al., 2021). The exact mechanism of how this is initiated is not known. One of the common features shared between neurodegeneration and COVID-19 is age. Age is the major risk factor for neurodegenerative diseases, while at the same time, older patients present with more severe symptoms and prolonged course of COVID-19 (Verkhratsky et al., 2020).

There are several explanations, how SARS-CoV-2 virus could contribute to neurodegeneration. The virus can directly invade the brain *via* the olfactory bulb, retrograde axonal transport from peripheral nerve endings, *via* hematogenous or lymphatic routes, and across the blood–brain barrier (Dolatshahi et al., 2021; Krasemann et al., 2022). Infection of the neurons combined with peripheral leukocyte activation results in the upregulation of pro-inflammatory cytokines, leading to neurodegenerative changes related to neuroinflammation (Verkhratsky et al., 2020; Dolatshahi et al., 2021; Haidar et al., 2021; Rosen et al., 2021). In severe cases, systemic inflammation can cause acute brain damage associated with psychiatric symptoms and cognitive impairment which is indicative of neurodegeneration (Verkhratsky et al., 2020). Furthermore, acute respiratory distress syndrome and sepsis often seen in COVID-19 induce hypoxemia and hypoperfusion, leading to oxidative stress and neurodegeneration (Verkhratsky et al., 2020; Dolatshahi et al., 2021; Haidar et al., 2021; Rosen et al., 2021). All of the above implies that SARS-CoV-2 is capable of entering the CNS and causing neurodegeneration.

The prevalence of neurodegenerative disorders is increasing, mostly due to extensions in lifespan, but nowadays COVID-19 may be a contributing factor as well. Several neurodegenerative diseases such as Alzheimer’s disease (AD), Parkinson’s disease (PD), and multiple sclerosis (MS) have already been associated with COVID-19, both in terms of development and in terms of worse disease prognosis (Ferini-Strambi and Salsone, 2021; Hu et al., 2021). Although information about the link between Huntington’s disease (HD) or amyotrophic lateral sclerosis (ALS) and COVID-19 is still scarce, some reports indicating that the virus affects the disease diagnosis, prognosis and patient care, have been published (Hu et al., 2021; Li and Bedlack, 2021; Vavougios, 2021). Long before COVID-19 pandemics, it was reported that antibodies against coronaviruses can be found in the cerebrospinal fluid of PD patients (Fazzini et al., 1992). Coronaviruses can enter the brain through the nasal cavity causing anosmia or hyposmia (Antonini et al., 2020). The latter is one of the main prodromal features of PD and the α-synuclein deposition in the olfactory bulb, among other locations, is one of the main pathological hallmarks (Kalia and Lang, 2015; Ferini-Strambi and Salsone, 2021), which indicates a strong association between PD and COVID-19. Furthermore, some COVID-19 patients develop cognitive deficits after the primary infection, indicating, that there might be a link between COVID-19 infection and dementia pathogenesis (Ye et al., 2020; Douaud et al., 2022). MS may also occur in association with COVID-19 infection as there were case reports published reporting the MS-like demyelination after the SARS-CoV-2 infection (Ismail and Salama, 2022).

Relation between COVID-19 and neurodegeneration has been shown in clinical setting observing a simultaneous or sequential development of COVID-19 and neurodegeneration, but perturbations on molecular level have been detected as well. A proteome study described the interactions of SARS-CoV-2 proteins with human proteins from several aging-related pathways, such as vesicle trafficking (NSP6, NSP7, NSP10, NSP13, NSP15, ORF3A, E, and ORF8), lipid modifications (Spike), RNA processing and regulation (NSP8, N), ubiquitin ligases (ORF10), and mitochondrial activity (NSP4, NSP8, and ORF9C; Gordon et al., 2020; Lippi et al., 2020). Moreover, infections with RNA viruses, also SARS-CoV-2 in particular, were already associated with increased production of α-synuclein (Bouali-Benazzouz and Benazzouz, 2021; Rosen et al., 2021) and amyloid-β (Aβ; Sun et al., 2021). It has also been shown, that SARS-CoV-2 infection could facilitate the spread of aggregated tau protein *via* the secretion of extracellular vesicles (Liu et al., 2021). Furthermore, it was reported that the transcriptomic perturbations are shared between COVID-19 and HD (Vavougios, 2021). These findings indicate a strong molecular link between SARS-CoV-2 and neurodegeneration, in particular PD and AD as two most common neurodegenerative disorders.

MiRNAs are the most studied small noncoding RNAs. They are functional RNA molecules of approximately 22 nucleotides in length that lack protein-coding properties (Siedlecki-Wullich et al., 2021). MiRNAs regulate gene expression on the post-transcriptional level. They bind to the 3-untranslated region of the target messenger RNA (mRNA) by a partially complementary sequence. A single miRNA can bind hundreds of different mRNA targets. The most frequent consequence of miRNA binding is translational repression (Filipowicz et al., 2008; Daugaard and Hansen, 2017).

Many miRNAs have already been associated with neurodegenerative pathologies and COVID-19 as biomarkers of the disease, either in terms of susceptibility or disease prognosis (Ravnik-Glavač and Glavač, 2020; Dong and Cong, 2021; Kuo et al., 2021; Pietrasik et al., 2021; Siedlecki-Wullich et al., 2021; Paul et al., 2022), but the overlap between COVID-19 and neurodegenerative diseases is unknown. Several miRNAs were repeatedly dysregulated in various neurodegenerative diseases, which functionally overlap in pathways related to Aβ genesis, regulation of AMPA receptor subunits, autophagy homeostasis, apoptosis, microglial activation, blood brain barrier maintenance, and neurogenesis (Juźwik et al., 2019). miRNAs involved in immune system regulation, more specifically NF-κB signaling, were frequently observed to be dysregulated in neurodegeneration processes as well (Juźwik et al., 2019). Such miRNAs involved in both immune and nervous system are called neurimmiRs and have already been suggested as potential drug targets in neurodegeneration (Soreq and Wolf, 2011). Additionally, altered miRNA levels have been associated with the formation of reactive oxygen species (ROS) and mitochondrial dysfunction, which are both hallmarks of neurodegenerative diseases (Catanesi et al., 2020). Similarly, many miRNAs are dysregulated in COVID-19 as it was shown that SARS-CoV-2 infection significantly alters plasma miRNA composition from an early stage of COVID-19 (Fernández-Pato et al., 2022).

Given the fact that miRNA seem to have a great potential as biomarkers of both neurodegenerative diseases and COVID-19, and that COVID-19 patients can present with clinical signs of neurodegeneration, we explored, whether miRNA could provide information on the shared pathways between neurodegeneration and COVID-19 pathogenesis. We have first performed a thorough literature screening and database mining to find the shared miRNA landscapes of SARS-CoV-2 and neurodegeneration. Next, we also identified shared pathways between the two pathologies and listed top miRNA candidates that could potentially serve as biomarkers of neurodegeneration in COVID-19 patients or as valuable treatment targets or agents to halt or prevent neurodegeneration in COVID-19 patients.

## 2. Methods

Literature screening approach and database mining were combined in search for common miRNAs shared between neurodegenerative diseases and COVID-19. Pathway enrichment analysis was performed on the list of target genes of the overlapping miRNAs.

First, differentially expressed miRNA in body fluids or tissues of COVID-19 patients were extracted from literature. PubMed search for original articles was performed using broad keyword string “COVID-19 and miRNA” to include as many relevant articles as possible. PRISMA flow diagram is presented in the Supplementary Figure S1 (Additional file 1). Following exclusion criteria, articles evaluating viral miRNAs, *in vitro* or *in silico* analyses, articles overlapping with other diseases and review articles, commentaries, preprints or articles not in English were not included in further analysis. The two authors (SRT and DV) independently reviewed all abstracts and articles to evaluate whether the study would meet criteria for inclusion in the analysis. If there was any disagreement between the authors about the inclusion of a study, a third author (KG) reviewed the study and made a decision. Furthermore, a list of miRNAs, associated with five most prevalent neurodegenerative diseases (AD, PD, MS, ALS and HD), was obtained from The Human microRNA Disease Database (HMDD, accessed on 21 .1. 2022; Huang et al., 2019). As HMDD reports only mature miRNAs (without specifying the-5p or-3p forms), we used mature miRNA forms also for the COVID-19 miRNA list. Key miRNAs were defined as miRNAs associated with both COVID-19 and all five neurodegenerative diseases and miRNAs deregulated in at least four COVID-19 studies with the same direction of effect (up-or down-regulated) and also deregulated in at least one neurodegenerative disease.

**Figure 1:**
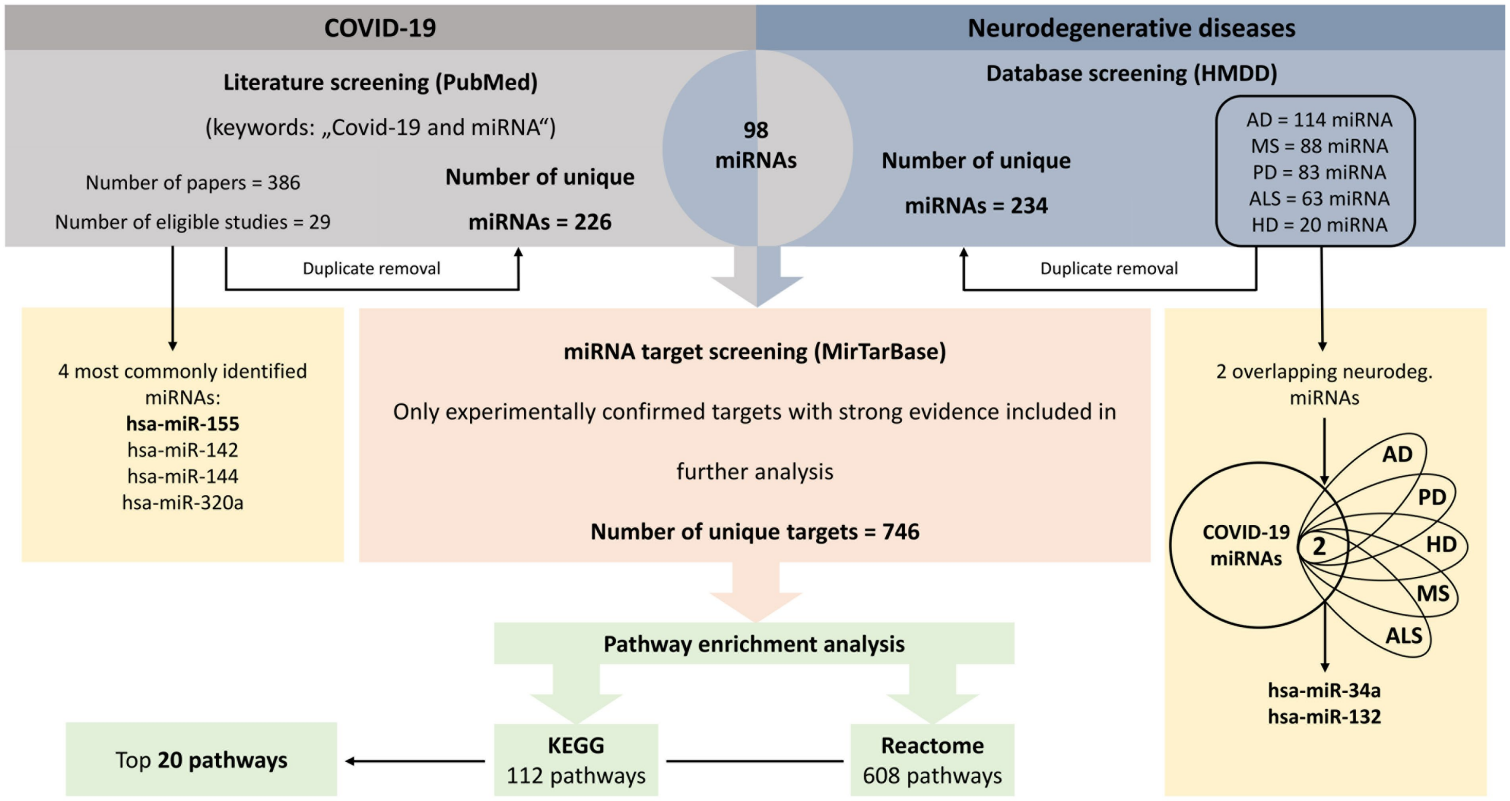
Workflow of the analysis. Integration of literature and database screening to identify miRNAs associated with neurodegenerative diseases and COVID-19, their target genes and pathway enrichment analysis. AD, Alzheimer’s disease; ALS, amyotrophic lateral sclerosis; COVID-19, coronavirus disease 2019; HD, Huntington’s disease; HMDD, Human microRNA Disease Database; MS, multiple sclerosis; PD, Parkinson’s disease.

In search for overlapping miRNAs (Figure 1), a list of unique COVID-19 miRNAs was then compared to a list of unique neurodegenerative diseases’ miRNAs. Venn diagrams were generated using Bioinformatics & Evolutionary Genomics group webtool (accessed on 12. 2. 2022; UGent, n.d.).

Next, common miRNAs were screened for gene targets using miRTarBase (accessed on 11. 2. 2022; Huang et al., 2020). We chose miRTarBase due to the fact that it allows to screen for experimentally confirmed target genes with all three strong evidence generating methods (Reporter assay, Western blot and qPCR). Only those target genes were included in further analysis (Figure 1). However, we were not able to differentiate whether a miRNA is up-or down-regulated in COVID-19 patients, because different studies reported different directions of effect.

A list of identified unique target genes served as a template for pathway enrichment analysis (Figure 1). Two different approaches were used; Kyoto Encyclopedia of Genes and Genomes (KEGG) enrichment was performed using The Database for Annotation, Visualization and Integrated Discovery (DAVID) Functional annotation tool (accessed on 11. 2. 2022; Huang da et al., 2009a,b), while Reactome (accessed on 12. 2. 2022) was used for Reactome pathway enrichment (Sidiropoulos et al., 2017). *p*-values below 0.05 after false discovery rate (FDR) adjustment were considered statistically significant. Top twenty significant pathways were visualized with bubble plots using GraphPad Prism 9 (GraphPad Software, San Diego, CA, USA).

## 3. Results

Between March 2020 and 20^th^ January 2022, 29 articles investigated miRNA expression changes associated with SARS-CoV-2 infection, mostly in plasma, serum or blood cell samples. In total, 226 unique miRNAs were identified, while 62 (27.4%) miRNAs were reported in more than one study. In patients with COVID-19, 108 (47.8%) miRNAs were down-regulated, 78 (34.5%) were up-regulated and 40 (17.7%) were either up- or down-regulated (Table 1). The most commonly identified miRNAs were hsa-miR-142, which was down-regulated in 3 studies (5p form) and up-regulated in 2 studies (3p form in 1 study, 5p in 1), hsa-miR-144, which was down-regulated in 3 studies (3p form in 2 studies, 5p in 1) and up-regulated in 2 studies (both 3p and 5p form in 1 study), and hsa-miR-320a, which was down-regulated in 2 studies (3p form in 1 study) and up-regulated in 3 studies (3p form in 2 studies). Additionally, hsa-miR-155 was upregulated in 4 studies. Among these, only hsa-miR-155 showed consistent direction of effect across the studies, which is why we highlighted it as a key miRNA.

**Table 1:** miRNAs with changed expression levels in COVID-19.

Reference	Disease stages	Sample type	miRNA					
			Up-regulated miRNA			Down-regulated miRNA		
Tang et al. (2020)	Moderate vs.severe vs. HC	Red blood cell depleted whole blood	hsa-miR-3605-3p	hsa-miR-486-3p		hsa-miR-146a-5p	hsa-miR-31-5p	hsa-miR-342-3p	hsa-miR-15b-5p	hsa-miR-18a-3p	hsa-miR-486-5p	hsa-miR-21-5p	hsa-miR-99a-5p	hsa-miR-181a-2-3p				hsa-miR-142-5p		
	Li et al. (2020)		Mild + moderate vs. HC	Peripheral blood		hsa-miR-16-2-3p	hsa-miR-6501-5p	hsa-miR-618	hsa-miR-183-5p	hsa-miR-627-5p	hsa-miR-144-3p
	Centa et al. (2020)		Severe vs. HC	*Post mortem* lung biopsies		/	hsa-miR-26a-5p	hsa-miR-29b-3p	hsa-miR-34a-5p
Zheng et al. (2020)	Mild vs. moderate vs. severe	Peripheral blood mononuclear cells	hsa-miR-100-5p	hsa-miR-26a-5p	hsa-miR-374b-5p	hsa-let-7e-5p	hsa-miR-328-3p	hsa-miR-671-3p
			hsa-miR-10395-3p	hsa-miR-26b-5p	hsa-miR-374c-5p	hsa-miR-10399-3p	hsa-miR-3615	hsa-miR-6772-3p
			hsa-miR-138-5p	hsa-miR-32-5p	hsa-miR-376a-3p	hsa-miR-10a-5p	hsa-miR-423-5p	hsa-miR-6842-3p
			hsa-miR-144-5p	hsa-miR-338-3p	hsa-miR-4,772-5p	hsa-miR-11400	hsa-miR-423-3p	hsa-miR-7706
			hsa-miR-144-3p	hsa-miR-33a-3p	hsa-miR-4,791	hsa-miR-1249-5p	hsa-miR-424-3p	hsa-miR-7977
			hsa-miR-181c-5p	hsa-miR-708-5p	hsa-miR-514a-3p	hsa-miR-1273 h-3p	hsa-miR-4646-3p	hsa-miR-877-5p
			hsa-miR-186-5p	hsa-miR-95-3p	hsa-miR-548w	hsa-miR-130b-5p	hsa-miR-4659b-3p	hsa-miR-92b-3p
			hsa-miR-6718-5p	hsa-miR-374a-5p		hsa-miR-139-5p	hsa-miR-484	hsa-miR-941-1
						hsa-miR-139-3p	hsa-miR-490-3p	hsa-miR-941-2
						hsa-miR-145-5p	hsa-miR-5193	hsa-miR-941-3
						hsa-miR-1908-5p	hsa-miR-584-5p	hsa-miR-941-4
						hsa-miR-195-3p	hsa-miR-625-3p	hsa-miR-941-5
						hsa-miR-320a-3p	hsa-miR-6501-5p	hsa-miR-942-5p
Sabbatinelli et al. (2021)	Tocilizumab treatment vs. HC	Serum	/			hsa-miR-146a-5p		
Garg et al. (2021)	Mechanically ventilated vs. HC	Serum	hsa-miR-21	hsa-miR-208a	hsa-miR-499	/
			hsa-miR-155					
Bagheri-Hosseinabadi et al. (2021)	COVID-19 vs. HC	Blood	/			hsa-miR-10b		
Mi et al. (2021)	Osteogenicdifferentiation in COVID-19	Blood	hsa-miR-4485			/		
	Yang et al. (2021)								COVID-19 vs. HC	Plasma	/			hsa-miR-451a		
Li et al. (2021)	COVID-19 vs. HC	Blood	hsa-miR-16-2-3p	hsa-miR-6501-5p	hsa-miR-505-5p	hsa-miR-183-5p	hsa-miR-4521	hsa-miR-18b-5p
			hsa-miR-5695	hsa-miR-4659a-3p	hsa-miR-125b-5p	hsa-miR-627-5p	hsa-miR-144-3p	hsa-miR-3613-5p
			hsa-miR-10399-3p	hsa-miR-142-5p	hsa-miR-618	hsa-miR-21-5p	hsa-miR-199a-3p	hsa-miR-29b-2-5p
						hsa-miR-20a-5p	hsa-miR-199b-3p	hsa-miR-32-5p
						hsa-miR-18a-5p	hsa-miR-96-5p	
Keikha et al. (2021)	COVID-19 (grades 1–5) vs. HC	Blood	hsa-miR-17-3p	hsa-miR-31-3p	hsa-miR-29a-3p	hsa-miR-126-3p
Saulle et al. (2021)	COVID-19 pregnant vs. uninfected pregnant women	Blood	hsa-miR-21	hsa-miR-29c	hsa-miR-155	/
			hsa-miR-23b	hsa-miR-98	hsa-miR-150	
			hsa-miR-28	hsa-miR-326	hsa-miR-146	
			hsa-miR-29a	hsa-miR-17		
			hsa-miR-92	hsa-miR-223		
Farr et al. (2021)	Moderate vs. severe vs. HC	Plasma	hsa-let-7e-5p	hsa-miR-1290	hsa-miR-4,742-3p	hsa-miR-766-3p	hsa-miR-5189-3p	hsa-miR-3913-5p
			hsa-miR-195-5p	hsa-miR-103a-3p	hsa-miR-423-5p	hsa-miR-651-5p	hsa-miR-6772-3p	hsa-miR-28-5p
			hsa-let-7a-5p	hsa-miR-320b	hsa-miR-320c	hsa-miR-1275	hsa-miR-145-3p	hsa-miR-551b-3p
			hsa-miR-483-5p	hsa-miR-193a-5p	hsa-miR-142-3p	hsa-miR-3198	hsa-miR-4772-3p	hsa-let-7i-3p
			hsa-miR-3125	hsa-miR-206	hsa-miR-92a-3p	hsa-miR-627-5p	hsa-miR-1226-3p	hsa-miR-548 k
			hsa-miR-30a-5p	hsa-miR-148a-3p	hsa-miR-6,721-5p	hsa-miR-4662a-5p	hsa-miR-589-3p	hsa-miR-18a-3p
			hsa-miR-27a-5p	hsa-miR-320a-3p	hsa-let-7f-5p	hsa-miR-3684	hsa-miR-210-3p	hsa-miR-491-5p
			hsa-miR-2116-3p	hsa-miR-576-5p		hsa-miR-3617-5p	hsa-miR-3115	hsa-miR-6503-3p
			hsa-miR-31-5p	hsa-miR-197-3p		hsa-miR-500b-3p	hsa-miR-769-3p	hsa-miR-3065-3p
						hsa-miR-664b-3p	hsa-miR-873-5p	hsa-miR-150-5p
Donyavi et al. (2021)	Acute and post-acute COVID-19 vs. HC	Peripheral blood mononuclear cells	hsa-let-7b-3p	hsa-miR-146a-3p	hsa-miR-29a-3p	/
			hsa-miR-155-5p					
Grehl et al. (2021)	Mild vs. severe	Plasma	hsa-miR-4,516	hsa-miR-320b	hsa-miR-629-5p	hsa-miR-454-3p	hsa-miR-126-3p	hsa-miR-342-3p
			hsa-miR-362-5p	hsa-miR-320c	hsa-miR-1180-3p	hsa-miR-625-3p	hsa-miR-146b-5p	hsa-miR-193b-3p
			hsa-miR-548 k	hsa-miR-320d	hsa-miR-502-3p	hsa-miR-30b-5p	hsa-miR-30c-5p	hsa-miR-190a-5p
			hsa-miR-320a-3p	hsa-miR-185-5p		hsa-miR-192-5p	hsa-miR-144-5p	hsa-miR-365b-3p
						hsa-miR-451a	hsa-miR-29a-3p	hsa-miR-122b-5p
						hsa-miR-197-3p	hsa-miR-363-3p	hsa-miR-122-3p
						hsa-miR-29b-3p	hsa-miR-99a-5p	
Chen et al. (2021)	COVID-19 vs. HC	Peripheral blood mononuclear cells	hsa-miR-485-5p	hsa-miR-3614-5p	hsa-let-7d-3p	hsa-miR-548ar-3p	hsa-miR-514a-3p	hsa-miR-4521
			hsa-miR-1226-3p	hsa-miR-103a-2-5p	hsa-miR-652-3p	hsa-miR-125b-1-3p	hsa-miR-615-3p	hsa-miR-142-5p
						hsa-miR-196a-5p	hsa-miR-184	hsa-miR-1291
						hsa-miR-34c-5p	hsa-miR-192-5p	hsa-miR-4772-5p
						hsa-miR-708-3p	hsa-miR-499a-5p	hsa-miR-141-3p
						hsa-miR-27a-3p	hsa-miR-340-3p	hsa-miR-3615
						hsa-miR-642a-5p	hsa-miR-581	hsa-miR-659-5p
						hsa-miR-509-3-5p	hsa-miR-3194-3p	hsa-miR-132-5p
							hsa-miR-511-5p	hsa-miR-4473
Pimenta et al. (2021)	Non-hospitalized vs. hospitalized vs. severe vs. HC	Saliva	hsa-miR-200c-3p			/		
de Gonzalo-Calvo et al. (2021)	Hospitalized vs. ICUsurvivor vs. non-survivor	Plasma	hsa-miR-27a-3p	hsa-miR-148a-3p	hsa-miR-491-5p	hsa-miR-16-5p	hsa-miR-150-5p	hsa-miR-486-5p	hsa-miR-27b-3p	hsa-miR-199a-5p		hsa-miR-92a-3p	hsa-miR-451a	
	Fayyad-Kazan et al. (2021)		COVID-19 vs.HC	Plasma		hsa-miR-15a-5p	hsa-miR-19b-3p		hsa-miR-92a-3p	hsa-miR-17-5p	hsa-miR-142-5p	
hsa-miR-19a-3p		hsa-miR-23a-3p			hsa-miR-320a							
McDonald et al. (2021)	COVID-19 vs. HC	Serum and urine	hsa-miR-2392			/		
Haroun et al. (2022)	COVID-19 vs. HC	Plasma	hsa-miR-155			/		
Agwa et al. (2021)	Mild vs. severe vs. HC	Blood	hsa-miR-4257			/		
Gutmann et al. (2022)	Non-severe vs. severe vs. HC	Plasma	hsa-miR-122	hsa-miR-133a		/		
	Mild vs. moderate vs. severe									
Wu et al. (2021)	COVID-19 vs. HC	Plasma	hsa-miR-29b-3p	hsa-miR-1246		hsa-miR-186-5p	hsa-miR-15a-5p	
Meidert et al. (2021)	Mild vs. severe vs. HC	Extracellular vesicles from serum	hsa-miR-193a-5p	hsa-miR-197-3p	hsa-miR-206	/		
			hsa-let-7 g-5p	hsa-miR-20a-5p					
Akula and Bolin (2022)	Moderate+severe vs. HC	Blood	hsa-miR-3197	hsa-miR-4690-5p	hsa-miR-1915-3p	hsa-miR-150-5p	hsa-miR-122-5p	hsa-miR-494-3p
			hsa-miR-3652						hsa-miR-375		
Liu et al. (2022)	Moderate vs. severe vs. HC	Blood	hsa-miR-130a-3p	hsa-miR-29b-3p		/		
Parray et al. (2021)	Asymptomatic vs. mild vs. severe	Blood	hsa-miR-1246	hsa-miR-3609	hsa-miR-3651	hsa-miR-3180-3p	hsa-miR-6805-5p	hsa-miR-8073
			hsa-miR-4532	hsa-miR-199a-5p	hsa-miR-1273 h-3p	hsa-let-7i-5p	hsa-miR-98-5p	hsa-miR-4750-5p
			hsa-miR-145-5p	hsa-miR-139-5p		hsa-miR-4632-5p	hsa-miR-3185	hsa-miR-6075
			hsa-miR-3651	hsa-miR-145-5p		hsa-miR-6861-5p	hsa-miR-572	hsa-let-7i-5p
						hsa-miR-6802-5p	hsa-miR-371b-5p	hsa-miR-1231
						hsa-miR-5196-5p	hsa-miR-3180	hsa-miR-885-3p
						hsa-miR-92b-5p		
Duecker et al. (2021)	Moderate vs. severe vs. HC	Blood		/		hsa-miR-320a	hsa-miR-320b	hsa-miR-320c

**Figure 2:**
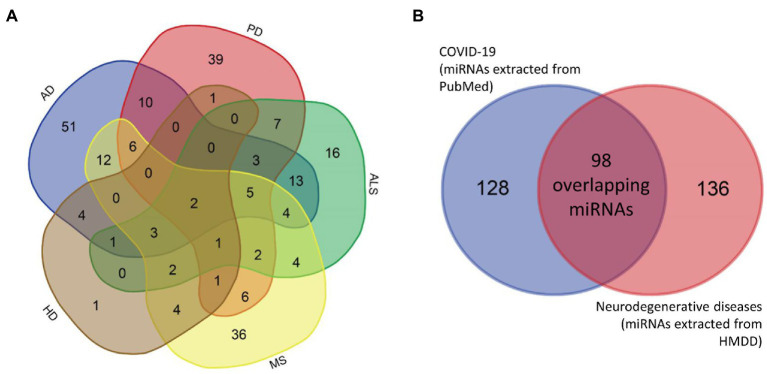
Venn diagrams. Venn diagrams showing the overlap between miRNAs associated with different neurodegenerative diseases separately **(A)** and the overlap between miRNAs associated with at least one neurodegenerative disease and COVID-19 **(B)**. AD, Alzheimer’s disease; ALS, amyotrophic lateral sclerosis; COVID-19, coronavirus disease 2019; HD, Huntington’s disease; MS, multiple sclerosis; PD, Parkinson’s disease.

Based on HMDD data, 114 unique miRNAs were dysregulated in AD, 88 miRNAs in MS, 83 miRNAs in PD, 20 miRNAs in HD and 63 miRNAs in ALS (Additional file 2: Supplementary Tables S1a–e). The overlap of miRNAs between different neurodegenerative diseases is presented in a Venn diagram in Figure 2A. In total, 234 unique miRNAs were associated with at least one neurodegenerative disease.

Comparison between neurodegenerative disease- and COVID-19-related miRNAs identified 98 common differentially expressed miRNAs (Figure 2B). Only two miRNAs, hsa-miR-34a and hsa-miR-132, were associated with all five investigated neurodegenerative disorders. Consequently, we added these two miRNAs to the list of key miRNAs.

For all 98 common miRNAs associated with at least one neurodegenerative disease and COVID-19, experimentally confirmed target genes were extracted from miRTarBase (Additional file 3: Supplementary Table S2). Screening resulted in 746 unique target genes confirmed with strong evidence. Target genes for common miRNAs hsa-miR-34a (47 genes) and hsa-miR-132 (10 genes) are presented in Table 2.

**Table 2:** Experimentally confirmed hsa-miR-34a and hsa-miR-132 target genes, associated with five investigated neurodegenerative disorders and COVID-19.

miRNA	Target genes
hsa-miR-34a-3p	*LDHA*
hsa-miR-34a-5p	*AXL*, *BCL2*, *BIRC5*, *CCND1*, *CD44*, *CDK4*, *CDK6*, *DLL1*, *E2F3*, *FOSL1*, *FUT8*, *GALNT7*, *GAS1*, *GFRA3*, *HDAC1*, *HNF4A*, *HOTAIR*, *INHBB*, *JAG1*, *KDM4A*, *KLF12*, *KLF4*, *L1CAM*, *MAGEA12*, *MAGEA2*, *MAGEA3*, *MAGEA6*, *MAP2K1*, *MAP3K9*, *MDM4*, *MET*, *MYB*, *MYC*, *MYCN*, *NOTCH1*, *NOTCH2*, *PDGFRA*, *PDGFRB*, *POU5F1*, *RICTOR*, *SIRT1*, *SIRT7*, *SNAI1*, *SRC*, *TCF7*, *YY1*
hsa-miR-132-3p	*ARHGAP32*, *CDKN1A*, *EGFR*, *FOXO1*, *HBEGF*, *HN1*, *KLHL11*, *MAPK1*, *RAF1*, *RB1*

**Figure 3:**
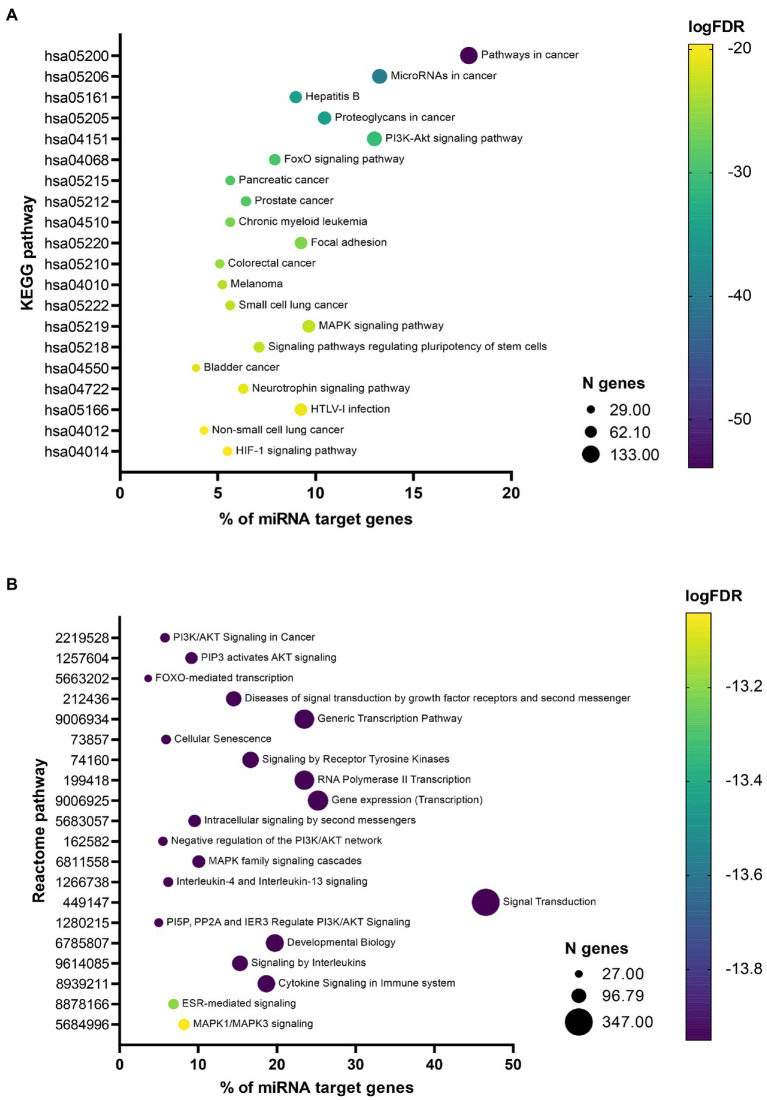
Bubble plots showing most significant enriched pathways. Bubble plots showing 20 most significant enriched Kyoto Encyclopedia of Genes and Genomes (KEGG) **(A)** and Reactome **(B)** pathways. The x-axis represents the percentage of 746 miRNA target genes associated with a specific pathway represented on the y-axis. Size of the bubble represents the number of genes associated with a specific pathway and the color of the bubble reflects the *p*-value after false discovery rate (FDR) adjustment.

Pathway enrichment analysis revealed, that miRNA target genes were significantly enriched (FDR < 0.05) in 112 KEGG pathways (Additional file 4: Supplementary Table S3a) and 608 Reactome pathways (Additional file 4: Supplementary Table S3b). Among the 20 most significant KEGG pathways (Figure 3A), 11 (55%) were associated with cancer, 6 (30%) with signaling, and 2 (10%) with infections and 1 (5%) with other biological processes. Among the 20 most significant Reactome pathways (Figure 3B), 10 (50%) were associated with signaling, 4 (20%) with transcription, 3 (15%) with immune system, 1 (5%) with cancer and 2 (10%) with other biological processes. Their association with neuroinflammation, neuroprotection, and cell senescence is further described in the discussion.

## 4. Discussion

Among miRNAs, dysregulated in patients with COVID-19, almost half were previously associated with at least one neurodegenerative disease. Three key miRNAs were identified as potential biomarkers of neurodegeneration in COVID-19: hsa-miR-34a, hsa-miR-132, and hsa-miR-155. Target genes of all shared miRNAs were enriched in several pathways associated with neurodegeneration, including neuroinflammation promoting signaling pathways, pathways related to neuroinflammation mediators, neuroprotective pathways, and cellular senescence pathway.

### 4.1. Interplay between COVID-19 and neurodegenerative diseases: Key miRNAs

Downregulation of hsa-miR-34a and hsa-miR-132 was associated with COVID-19 and all five investigated neurodegenerative diseases, whereas hsa-miR-155 was found to be upregulated in at least four COVID-19 expression studies and was also associated with several of the investigated neurodegenerative diseases. All listed COVID-19-related miRNAs were identified in human studies, whereas not all neurodegeneration-related miRNAs were identified in human samples. According to the HMDD, all three listed key miRNAs were confirmed to be associated with AD and MS in human studies. Furthermore, hsa-miR-132 was associated with PD and ALS in patients, while hsa-miR-34a was associated with ALS in patients as well. Other associations between one of the key miRNAs and a certain neurodegenerative disease were identified in other types of models. Expression of miRNA is spatiotemporally specific. It is affected by various factors, such as age, comorbidities, SARS-CoV-2 strain, COVID-19 vaccination status, time from SARS-CoV-2 infection, etc.(Martínez-Fleta et al., 2021) The choice of tissue for expression analysis and also taking into account the listed factors is thus important. However, considering that COVID-19 may induce a systemic inflammatory response, which relates to disease severity (Ferrara and Vitiello, 2021; Ramos-Casals et al., 2021), choosing plasma as a readily accessible reservoir of miRNAs originating from different tissues is reasonable. In body fluids, signaling molecules, including miRNAs, have been found as extracellular vesicle’s (EV) cargo. Understanding the role of EVs as important mediators of intercellular communication, their potential in miRNA associated gene regulation of COVID-19 patients has previously been studied (Meidert et al., 2021; Wang Y. et al., 2021). Furthermore, another potential mechanism for EV transfer to periphery can be associated with the breakdown of blood brain barrier, an overlapping feature of many neurodegenerative diseases and COVID-19 as well (Hill, 2019; Bonetto et al., 2022). To date, different miRNAs of neuronal origin have been found in blood plasma samples (Serpente et al., 2020; Bonetto et al., 2022).

Of note, we considered the identified miRNAs only as potential biomarkers of a certain phenotype, e.g., neurodegeneration in COVID-19 patients, meaning that they do not necessarily have to be of neuronal origin. However, we speculate that COVID-19 has similar effects on miRNA expression in both CNS and peripheral tissues. Studies on patients or human cell lines are emphasized with the hsa- prefix, whereas no prefix indicates different animal models.

#### 4.1.1. Hsa-miR-34a

Hsa-miR-34a is one of the miRNAs associated with COVID-19 and all neurodegenerative diseases investigated in our study. Relative expression of hsa-miR-34a-5p was significantly down-regulated in *post mortem* lung biopsies of patients that died of COVID-19 compared to controls that died of other causes (Centa et al., 2020).

Dysregulation of miR-34a was observed in ALS (Raheja et al., 2018; Rizzuti et al., 2018; Kmetzsch et al., 2021), PD (Grossi et al., 2021; Yang et al., 2022), AD (Sarkar et al., 2016; Cosín-Tomás et al., 2017; Li et al., 2020; Li and Cai, 2021), HD (Reynolds et al., 2018), and MS (Ghadiri et al., 2018). In AD, hsa-miR-34a was overexpressed in specific brain regions (Sarkar et al., 2016; Li and Cai, 2021), but downregulated in blood or CSF (Cosín-Tomás et al., 2017; Li et al., 2020). In PD, this miRNA was enriched in small EVs and associated with disease duration and clinical characteristics (Grossi et al., 2021). Hsa-miR-34a was also associated with disease relapse in MS (Ghadiri et al., 2018) and disease duration in ALS (Raheja et al., 2018), while upregulation in CSF of ALS patients was also observed (Rizzuti et al., 2022). It was proposed as a negative regulator of *C9ORF72*, a gene commonly mutated in familial ALS (Kmetzsch et al., 2021).

MiR-34a regulates multiple processes that can contribute to neurodegeneration, such as neuronal differentiation, synaptic plasticity, neurogenesis, cognitive function, brain aging and energy metabolism (Dickson et al., 2013; Sarkar et al., 2016; Grossi et al., 2021). Specifically, miR-34a can regulate several genes important for AD pathogenesis, such as sirtuin 1 (*SIRT1*), ADAM metallopeptidase domain 10 (*ADAM10*), triggering receptor expressed on myeloid cells 2 (*TREM2*), and BCL2 apoptosis regulator (*BCL2*; Nunomura and Perry, 2020). Hsa-miR-34a can directly decrease expression of tau protein, a key player in AD pathogenesis (Dickson et al., 2013). Studies have also shown that miR-34a affects Aβ induced neurotoxicity through regulation of beta-secretase 1 (*BACE1*; Li et al., 2020), as well as amyloid precursor protein (*APP*) processing and cognitive defects (Cosín-Tomás et al., 2017; Jian et al., 2017). Additionally, this miRNA was implicated in signaling pathways associated with endothelial dysfunction (Centa et al., 2020) and acting as a tumor suppressor in different types of cancer (Li et al., 2021). It may also affect ROS production and response to oxidative stress by regulating expression of nuclear factor erythroid 2-related factor 2 (NRF2, encoded by *NFE2L2*) and other genes (Climent et al., 2020; Nunomura and Perry, 2020). NRF2 is a transcription factor that plays an important role in various neurodegenerative diseases and was even proposed as a potential therapeutic target (Saha et al., 2021).

Hsa-miR-34a target genes are overrepresented in pathways associated with viral infectious diseases (Centa et al., 2020) and hsa-miR-34a was dysregulated in response to infection with various viruses, especially human papilloma virus and influenza A virus, where hsa-miR-34a was downregulated [reviewed in (Lv et al., 2019)]. Several hsa-miR-34a target genes have already been found to be differentially expressed in RNA sequencing studies or protein levels were found to be changed due to SARS-CoV-2 infection, such as *AXL* (Wang S. et al., 2021) *BCL2* (Lorente et al., 2021), *LDHA* (Xu et al., 2021), *SIRT1* (Bordoni et al., 2021; D'Agnillo et al., 2021), *PDGFRB* (Kamp et al., 2022), and *MYC* (Banaganapalli et al., 2021). AXL is involved in virus cell entry (Naik et al., 2022), while BCL2 is an apoptosis inhibitor (Yapasert et al., 2021). LDHA was upregulated on both mRNA (Qi et al., 2021) and protein (Xu et al., 2021) levels and shows potential as a distinguishing biomarker between healthy and SARS-CoV-2 infected people. Expression of SIRT1 mRNA, a major inhibitor of oxidative stress–induced senescence in lung endothelial and epithelial cells, was shown to be decreased in COVID-19 (D'Agnillo et al., 2021). All this shows, that downregulation of hsa-miR-34a in COVID-19 may be one of the factors contributing to the neurological complications in COVID-19 patients.

#### 4.1.2. Hsa-miR-132

Second miRNA associated with all investigated neurodegenerative diseases and COVID-19 was hsa-miR-132. Relative expression of hsa-miR-132-5p was significantly down-regulated in peripheral mononuclear cell samples of COVID-19 patients (Chen et al., 2021).

MiR-132 is one of the most commonly dysregulated miRNAs in neurodegenerative diseases, particularly in AD (Lau et al., 2013; Ma et al., 2014; Smith et al., 2015; Salta and De Strooper, 2017; El Fatimy et al., 2018; Cha et al., 2019; Li and Cai, 2021), but also PD (Alieva et al., 2015; Yang et al., 2019; Shu et al., 2020; Gong et al., 2022), ALS (Vrabec et al., 2018), MS (Miyazaki et al., 2014; Regev et al., 2017) and HD (Fukuoka et al., 2018). Hsa-miR-132 was consistently downregulated in AD, both in the brain and blood, including in neurally-derived EVs (Lagos et al., 2010; Smith et al., 2015; Pichler et al., 2017; Cha et al., 2019; Tasker et al., 2021). On the other hand, upregulation of hsa-miR-132 was mostly observed in PD, where it was also associated with disease duration and severity and dopaminergic neurodegeneration (Yang et al., 2019; Gong et al., 2022), but downregulation was also reported by some studies (Shu et al., 2020). Additionally, increased expression was observed after treatment (Alieva et al., 2015).

MiR-132 is one of the most abundant brain miRNAs and it was proposed as a neuroprotective miRNA that regulates multiple processes in the nervous system, such as neuronal differentiation, survival and migration, synaptic plasticity, neurogenesis, apoptosis, memory, and cognitive function (Smith et al., 2015; Salta and De Strooper, 2017; Zhang and Bian, 2021). Several miR-132 target genes were identified and many are also important in neurodegenerative diseases, such as *BACE1*, *APP*, *SIRT1*, acetylcholinesterase (*ACHE*), nitric oxide synthase 1 (*NOS1*), and mitogen-activated protein kinase (*MAPK1*; Miyazaki et al., 2014; Salta and De Strooper, 2017; Qu et al., 2021; Zhang and Bian, 2021). It also regulates signaling pathways, e.g., PI3K/AKT and Notch signaling (Salta and De Strooper, 2017). Importantly, miR-132 exerts a neuroprotective effect against Aβ and glutamate excitotoxicity and decreases tau protein expression (Dickson et al., 2013; Smith et al., 2015; El Fatimy et al., 2018; Varma-Doyle et al., 2021). Several studies have proposed that it could be a potential treatment target as restoration of miR-132 expression improved cognitive function in animal models (Qu et al., 2021; Zhang and Bian, 2021).

Hsa-miR-132 also plays an important role in inflammation and immune response (Salta and De Strooper, 2017). It represses inflammatory cytokines such as interleukin 1β (IL-1β), tumor necrosis factor (TNFα) and interleukin 6 (IL-6; Salta and De Strooper, 2017; Gong et al., 2022). Expression of hsa-miR-132 target genes, such as *MAPK1* (Hasankhani et al., 2021), and *FOXO1* (Jha et al., 2020), involved in neuroinflammation or neuroprotective mechanisms, respectively, was found to be affected in COVID-19 patients. Interestingly, hsa-miR-132 was previously upregulated in response to infection with different viruses (Lagos et al., 2010; Buggele et al., 2012; Qi et al., 2014; Zhang et al., 2019). Its upregulation decreased interferon response through interferon regulatory factor 1 (*IRF1*) in H1N1 influenza A virus infection (Zhang et al., 2019). It was speculated that miR-132 might be associated with nervous system complications such as encephalitis after varicella-zoster virus infection (Qi et al., 2014). All these findings suggest that hsa-miR-132 might be a factor contributing to neurological complications in COVID-19 patients and should be investigated in further studies.

#### 4.1.3. Hsa-miR-155

Hsa-miR-155 expression was upregulated in blood or peripheral blood mononuclear cells in four studies on COVID-19 (Donyavi et al., 2021; Garg et al., 2021; Saulle et al., 2021; Haroun et al., 2022). It was upregulated in both acute and post-acute patients and was further upregulated in severe or critically ill COVID-19 patients or patients that died (Donyavi et al., 2021; Garg et al., 2021; Haroun et al., 2022). It could also differentiate between COVID-19 and influenza-associated acute respiratory distress syndrome (Garg et al., 2021). Studies have therefore proposed hsa-miR-155 as a potential diagnostic biomarker for COVID-19 (Donyavi et al., 2021; Garg et al., 2021; Haroun et al., 2022).

Previous studies have found that miR-155 plays an important role in several neurodegenerative diseases: ALS (Cunha et al., 2018; Raheja et al., 2018), PD (Caggiu et al., 2018; Oliveira et al., 2020), AD (Song and Lee, 2015; Liang and Wang, 2021), and MS (Lopez-Ramirez et al., 2014; Ma et al., 2014; Niwald et al., 2017; Regev et al., 2017; Baulina et al., 2018). It was consistently upregulated in most studies on AD, PD, MS, ALS (Juźwik et al., 2019; Slota and Booth, 2019; Zingale et al., 2021). In PD, hsa-miR-155 was increased in patients treated with L-dopa and was proposed as a disease progression biomarker and potential treatment target (Caggiu et al., 2018). It was also associated with patients’ clinical characteristics in ALS and MS (Niwald et al., 2017; Raheja et al., 2018). Recently, in CSF EVs, miR-155 was specifically expressed in patients with encephalitis (Torii et al., 2022).

MiR-155 is widely regarded as a central proinflammatory miRNA in CNS, associated with numerous processes of neuroinflammation, including polarization, neurotoxicity, activation of different cell types and cell plasticity (Lopez-Ramirez et al., 2014; Gaudet et al., 2018; Slota and Booth, 2019). It could serve as a negative regulator of blood–brain barrier permeability through regulation of cell–cell complex molecules and focal adhesion components (Lopez-Ramirez et al., 2014). In different models, it was proposed as a potential therapeutic target in neurodegenerative diseases (Rastegar-Moghaddam et al., 2022).

Hsa-miR-155 is also abundantly expressed in B-cells, T-cells, macrophages, and dendritic cells (Zeng et al., 2015). It regulates T lymphocyte function: development and activation, cell–cell interaction and interferon γ signaling (Song and Lee, 2015). MiR-155 is associated with proinflammatory cytokines IL-1β, TNFα and IL-6, as well as auto-immunity (Lopez-Ramirez et al., 2014; Thome et al., 2016; Rastegar-Moghaddam et al., 2022). It was also associated with immune system disorders, infection, and cancer (Climent et al., 2020).

Among most important targets of hsa-miR-155 are suppressor of cytokine signaling 1 (*SOCS1*), inositol polyphosphate-5-phosphatase D (*SHIP1*), and interleukin 13 receptor subunit alpha 1 (*IL13RA1*; Gaudet et al., 2018; Slota and Booth, 2019; Zingale et al., 2021). In AD, it was also associated with AD-related genes such as phosphatidylinositol binding clathrin assembly protein (*PICALM*) and sortilin related receptor 1 (*SORL1*), and production of Aβ (Rastegar-Moghaddam et al., 2022). In PD, it was associated with Parkinsonism associated deglycase (*DJ-1*) and inflammatory responses induced by α-synuclein (Thome et al., 2016; Rastegar-Moghaddam et al., 2022). It was also associated with response to oxidative stress, NRF2 and inducible NO synthase (Climent et al., 2020).

A lot of studies have shown that hsa-miR-155 plays a crucial role in response to microbial infections and is upregulated in response to various viruses [reviewed in (Zeng et al., 2015; Jafarzadeh et al., 2021)]. Hsa-miR-155 is involved in both adaptive and innate antiviral immune response and it affects both inflammatory response and anti-virus response, e.g., viral clearance and persistence (Jafarzadeh et al., 2021). Hsa-miR-155 was also upregulated in viral infection of the CNS (Slota and Booth, 2019; Zingale et al., 2021). In coronavirus-induced neurologic disease model, miR-155 enhanced T cell trafficking to the CNS and was associated with host defence by regulating cytolytic activity and cytokine secretion (Dickey et al., 2016). In COVID-19 it was suggested to be associated with the signature cytokine storm (Donyavi et al., 2021). Importantly, this miRNA was associated with susceptibility to virus-induced neurologic disease such as encephalitis (Dickey et al., 2016). All these findings provide ample support that hsa-miR-155 regulation might be an important factor contributing to neurological complications in COVID-19 patients and should be investigated in further studies.

#### 4.1.4. Other miRNAs

Additionally, three miRNAs (hsa-miR-142, hsa-miR-144, and hsa-miR-320a) were dysregulated in blood or peripheral blood mononuclear cells in five studies on COVID-19 (Li et al., 2020; Tang et al., 2020; Zheng et al., 2020; Chen et al., 2021; Duecker et al., 2021; Farr et al., 2021; Fayyad-Kazan et al., 2021; Grehl et al., 2021; Li et al., 2021). Even though the reported direction of effect was not consistent among studies, further studies could provide more insight regarding the role of these miRNAs, as they were also often implicated in neurodegeneration.

MiR-142 downregulation promotes inflammatory processes and affects response to oxidative stress by regulating NRF2 expression (Sørensen et al., 2016; Regev et al., 2017; Tang et al., 2020; Varma-Doyle et al., 2021). It plays an important role in hematopoesis, inflammation, lung development, and response to viral infections (Shrestha et al., 2017). Changes in hsa-miR-142 expression were reported in ALS (Matamala et al., 2018), PD (Liu et al., 2019; Barbagallo et al., 2020), AD (Kumar et al., 2013; Sørensen et al., 2016; Song and Kim, 2017), and especially in MS (Ma et al., 2014; Regev et al., 2016, 2017, 2018; Mandolesi et al., 2017; Raheja et al., 2018; De Vito et al., 2022). It was also associated with disease progression in MS and ALS (Regev et al., 2016; Mandolesi et al., 2017; Regev et al., 2017; Matamala et al., 2018; Raheja et al., 2018; Regev et al., 2018; De Vito et al., 2022).

MiR-144 plays an important role in cancer, regulating cell proliferation, migration, apoptosis and other signaling pathways (Zhou et al., 2020). MiR-144 can also promote oxidative stress, as it regulates NRF2 in cooperation with hsa-miR-34a (Climent et al., 2020). It was identified as a negative regulator of host response to RNA viruses in mice models (Rosenberger et al., 2017). Hsa-miR-144 was dysregulated in ALS (Liguori et al., 2018; Raheja et al., 2018), PD (Xing et al., 2020; Zago et al., 2022), AD (Cheng et al., 2013), and MS (Roshani et al., 2021).

MiR-320a affects multiple processes associated with neurodegeneration and has been proposed as an important regulator of blood–brain barrier permeability (Sepramaniam et al., 2010; Aung et al., 2015). Altered hsa-miR-320a expression was observed especially in MS, where it was associated with disease relapse (Aung et al., 2015; Regev et al., 2016; Regev et al., 2018), but also in ALS and AD (Denk et al., 2018; Raheja et al., 2018; Tan et al., 2021). Interestingly, hsa-miR-320a was previously associated with adenoviral infection in lung cells (Zhao et al., 2015) and downregulated in HIV-1 patients suffering from mild cognitive impairment (Fatima et al., 2017).

### 4.2. Interplay between COVID-19 and neurodegenerative diseases: Key pathways

Our Reactome and KEGG pathway enrichment analyses highlighted some of the most significant pathways that could link COVID-19 and neurodegenerative diseases.

Overall, the most enriched pathways within the Reactome top 20 list belong to various signaling processes. Some of them are very broad and rather unspecific, such as »Diseases of signal transduction by growth factor receptors and second messengers«, »Signaling by receptor tyrosine kinases«, »Intracellular signaling by second messengers«, and »Signal transduction«. Additionally, three of the pathways are very general transcription-related pathways, such as »Generic transcription pathway«, »RNA Polymerase II transcription«, and »Gene expression (Transcription)«. Also the »Developmental biology« pathway according to the Reactome database (Sidiropoulos et al., 2017) presents processes taking place long before COVID-19 and its neurological consequences occur and is thus not further discussed. More than half of the pathways identified with the KEGG pathway enrichment analysis are associated with cancer (“Pathways in cancer,” “MicroRNAs in cancer,” “Proteoglycans in cancer,” “Pancreatic cancer,” “Prostate cancer,” “Chronic myeloid leukemia,” “Colorectal cancer,” “Melanoma,” “Small cell lung cancer,” “Bladder cancer” and “Non-small cell lung cancer”). This can indicate a strong overlap between COVID-19 disease and cancer pathways and can also have implications for cancer treatment (Zong et al., 2021). Additionally, two other pathways, “Focal adhesion” and “Signaling pathways regulating pluripotency of stem cells,” are very broad and unspecific and are thus not discussed in this paper.

However, several identified pathways show an important relationship between COVID-19 pathogenesis and neurodegeneration. A lot of these pathways indicate that neuroinflammation is the key shared process. Pivotal shared neuroinflammation promoting entities are pathways related to phosphatidyl-inositol-3-kinase (PI3K)/protein kinase B (AKT) signaling and mitogen-activated protein kinase (MAPK) signaling, whereas shared neuroprotective mechanisms belong to pathways related to the class O of forkhead box transcription factors (FOXO) signaling. Thus, these pathways present overlapping pathogenic processes and could serve as relevant drug targets for prevention of post-COVID-19 neurodegeneration. However, miRNAs found to be dysregulated in COVID-19 patients belonging to the same enriched pathway do not always present the same direction of effect. Thus, and also due to the fact that multiple miRNAs are involved in one pathway, it is not possible to state based on miRNA data whether the pathway is activated or inhibited in COVID-19.

#### 4.2.1. Neuroinflammation promoting signaling pathways

Inflammation is a feature of both, neurodegeneration and COVID-19. It has been proposed that weak chronic activation of neuroinflammation signaling pathways causes neurodegeneration (Chu et al., 2021; Li et al., 2021), whereas strongly activated neuroinflammation often causes acute disease progression similar to COVID-19 (Li et al., 2021). Furthermore, external inflammation, such as SARS-CoV-2 infection, and consequential immune signaling can both activate PI3K/AKT, MAPK, nuclear factor kappa-light-chain-enhancer of activated B cells (NF-κB), NLR family pyrin domain containing 3 (NLRP3), and mammalian target of rapamycin (mTOR) signaling as the core inflammation pathways (Li et al., 2021; Hensley et al., 2022; Khezri et al., 2022).

»PI3K/Akt signaling pathway in cancer« and “PI3K-Akt signaling pathway” were among the top enriched pathways in Reactome and KEGG top 20 lists, respectively. Under the umbrella of PI3K/AKT signaling we can also include »PIP3 activates AKT signaling« and »PI5P, PP2A and IER3 regulate PI3K/AKT signaling« pathways from Reactome analysis. The PI3K/AKT pathway is a key signaling cascade in cell biology and is highly involved in inflammation molecular functions. Activation of serine/threonine and tyrosine kinases through PI3K regulates different major cellular processes such as protein synthesis, apoptosis and cell proliferation (Franke et al., 1997; Khezri et al., 2022). Additionally, ROS response to SARS-CoV-2 spike in human bronchial and microvascular cells leads to activation of inflammation and apoptosis through PI3K/AKT pathway inhibition (Li et al., 2021). PI3K/Akt signaling specifically is over-activated in COVID-19 patients and could affect SARS-CoV-2 entry and replication in the host cells by regulating the clathrin-mediated endocytosis and glycolysis (Khezri et al., 2022). Thus, it also presents a potential drug target for COVID-19 treatment (Fattahi et al., 2022). The role of PI3K/AKT signaling in neurogenesis has also been proposed. Beneficial effect on survival of newly formed neurons during exercise elucidates the role of PI3K/AKT in neurogenesis and synaptic plasticity in a rat model (Bruel-Jungerman et al., 2009). Additionally, this pathway has already been associated with all five investigated neurodegenerative diseases (Mammana et al., 2018; Recabarren-Leiva and Alarcón, 2018; Chu et al., 2021). In the context of neurodegeneration, more specifically in AD, perturbations in PI3K/AKT pathway induce changes in action of fibrillary Aβ, insulin signaling, autophagy, oxidative stress defense, and neuroinflammation (Razani et al., 2021). A rare familial mutation in *PS1* can affect presenilin-1 (PS-1), an upstream PI3K regulator, and promote AD pathology by inhibiting PI3K/AKT signaling (Baki et al., 2004). Furthermore, phosphatase and tensin homolog (PTEN) induced PI3K/AKT pathway activation decreases endoplasmic reticulum stress response and apoptosis shown in an AD mouse model (Cui et al., 2017). PI3K/AKT signaling plays a crucial role in oxidative mechanisms in PD. Downregulation of PI3K/AKT, caused by SHC-transforming protein 3 (SHC3) silencing in PD rats can promote oxidative stress injury, leading to motor abnormalities (Gong et al., 2018). AKT-controlled inhibition of NAD(P)H dehydrogenase quinone 1 (NQO1), an important antioxidant system, aggravated PD pathogenesis in a rat model (Luo et al., 2019). Given that PI3K/AKT is important in both COVID-19 and neurodegenerative diseases, modulation of this pathway in specific cell types could present a relevant option for treatment of neurodegenerative diseases (Chu et al., 2021; Razani et al., 2021) and could open a window of opportunity to avoid neurodegenerative sequelae of COVID-19.

The MAPK signaling within two identified Reactome pathways (»MAPK family signaling cascades« and »MAPK1/MAPK3 signaling«) and one KEGG pathway (“MAPK signaling pathway”) may act either as a positive or negative regulator of viral replication. MAPK signaling integrates diverse stimuli signals and elicits multiple cellular responses, including cellular proliferation, differentiation, development, inflammatory responses and apoptosis (Zhang and Liu, 2002). The viral secretory proteins can trigger the extracellular signal-regulated kinase 1 and 2 (ERK1/2) activation, which is one of the MAP kinases (Mohanta et al., 2020). MAPK has been associated with viral entry mechanisms as well, since SARS-CoV-2 spike protein activates MAPK signaling through angiotensin II receptor type 1 (AT1) or transmembrane protease, serine 2 (TMPRSS2) upregulation (Patra et al., 2020; Cioccarelli et al., 2021). Furthermore, activation of the MAPK pathways due to SARS-CoV-2 infection contributes to higher cytokine levels and hyper-inflammatory responses (Peter et al., 2020). On proteomic level, MAPK was enriched in SARS-CoV-2 associated myocarditis as well (Weckbach et al., 2022). Similarly, MAP kinases, such as c-Jun N-terminal kinase (JNK), ERK1/2 and p38 are involved in creating the pro-inflammatory environment in age-related neurodegenerative diseases, which means that they support the cytokine release in neurodegenerative diseases as well. This uncontrolled release of cytokines may produce a self-perpetuating feedback loop enhancing inflammation and contributing to neuronal damage and death (Ahmed et al., 2020). There have been reports published stating the abnormal activation of MAP kinases in all five investigated neurodegenerative pathologies and MAPK has been linked to pathophysiological hallmarks of neurodegeneration (Albert-Gascó et al., 2020; Ten Bosch et al., 2021; Yadav et al., 2021). Important role of MAPK signaling in Aβ generation (Du et al., 2019) and cognitive decline independent of amyloid pathology has been proposed in AD (Johnson et al., 2022). Multiple levels of evidence support the TREM2-induced MAPK signaling in PD pathogenesis (Huang et al., 2021; Ruganzu et al., 2022). Microglial MAPK over-activity has been extensively studied in MS (Ten Bosch et al., 2021), while the therapeutic potential of MAPK pathway inhibition in demyelination recovery has also been proposed (Suo et al., 2019). Similarly, a therapeutic strategy for ALS, targeting MAPK-mediated axonal retrograde transport, has been studied (Gibbs et al., 2018). Based on these reports, we can conclude that dysregulation of the MAPK signaling due to SARS-CoV-2 infection could foster neurodegeneration in COVID-19 patients. In addition, MAPK signaling pathway also presents a potential druggable target to prevent or alleviate inflammation after COVID-19 and neurodegeneration.

#### 4.2.2. Pathways related to mediators of neuroinflammation

At least three further pathways indicate a strong overlap between neurodegeneration, COVID-19 and inflammation, such as »Interleukin-4 and interleukin-13 signaling«, »Cytokine signaling in immune system«, and »Signaling by interleukins«. The latter pathways greatly overlap with the enriched pathways described above. Recent findings suggest that COVID-19 could inflict structural and metabolic damage in the CNS by upregulating inflammatory cytokines. This phenomenon is known as the cytokine storm syndrome (Nagu et al., 2021), within which the glial cells induce neuroinflammation by the release of various pro-inflammatory cytokines, such as IL-6, TNFα, interleukin 5 (IL-5) and interleukin 2 (IL-2; Bohmwald et al., 2018). Among other inflammatory mediators, interleukin 10 (IL-10) has also been shown to be increased in COVID-19 (Savarraj et al., 2021). Recovered COVID-19 patients with long-term symptoms, mostly neurological complaints, have higher plasma levels of interleukin 4 (IL-4; Sun et al., 2021). This is an anti-inflammatory cytokine released as a response to neuroinflammation, trying to re-establish the neuronal homeostasis. However, the same study showed that plasma IL-6 levels present a more specific biomarker of neurological deficits after COVID-19 since elevation of IL-6 was observed specifically in neuro-long COVID-19 as compared to long COVID-19 without neurological symptoms (Sun et al., 2021). In general, SARS-CoV-2 infection leads to maladaptive innate immunity and overall inflammation with activated microglia and astrocytes contributing to neurodegenerative processes, including demyelination, blood brain barrier disruption, and aberrant activation of CNS (Gupta and Weaver, 2021). In chronically present neuroinflammatory environment glial cells are in charge of cytokine release and release of other pro-inflammatory mediators. Their secretome activates neighboring cells including other microglia and astrocytes, regardless of their encounter with pathogens or damage. Consequently, the neuroinflammation exacerbates and supports neurodegeneration (Ghosh et al., 2021). The role of interleukins and other cytokines in neurodegenerative diseases has been extensively studied. In meta-analyses, dysregulation of several cytokines was observed in blood or CSF samples. In particular, TNFα, IL-6 and IL-1β were consistently increased in AD, PD and ALS patients (Qin et al., 2016; Hu et al., 2017; Lai et al., 2017; Shen et al., 2019). Targeting specific interleukin release could potentially slow down the process of neurodegeneration in COVID-19 patients, especially later after their recovery.

#### 4.2.3. Neuroprotective pathways

A highly significant pathway according to our Reactome analysis is the »FOXO-mediated transcription« pathway. Interestingly, one of the prime regulators of FOXO activity is the PI3K/AKT pathway that was found to be enriched in our analysis as well. Generally, FOXO proteins promote neuronal health and viability (McLaughlin and Broihier, 2018). They regulate neuronal apoptosis in response to ROS accumulation and are important in regulating neural cell fate and function and essential for preventing age-dependent axonal degeneration (Santo and Paik, 2018). Aβ exposure in AD brain leads to FOXO3A dephosphorylation and mitochondrial dysfunction (Santo and Paik, 2018). Furthermore, moderate FOXO3 overexpression is protective in α-synuclein overexpressing dopaminergic neurons and drives α-synuclein into insoluble aggregates, suggesting that FOXO3 induces autophagy (McLaughlin and Broihier, 2018). The latter suggests that FOXO3A as a member of the FOXO family protects the brain against neurodegenerative assaults. FOXOs play an important role in response to viral infections as well. They upregulate various pro-inflammatory cytokines such as IL-1β and interleukin 9, Toll-like receptor (TLR) 1 and TLR4, etc., which not only regulate the host inflammatory response but also alter the innate immune response (Cheema et al., 2021). FOXO mediators regulate the expression of many different genes, among them also Kelch-like ECH-associated protein 1 (Keap1) and haem oxygenase 1 (HO-1), which both aim to ameliorate inflammation (Cheema et al., 2021). It has been proposed that repurposing FOXO activators for COVID-19 treatment (Cheema et al., 2021) could perhaps prevent or decrease neurodegenerative sequelae.

»ESR-mediated signaling« was identified as an overlapping pathway between neurodegeneration and COVID-19 as well. Estrogens can exert different neuroprotective roles by acting as antioxidants, promoting DNA repair, inducing expression of growth factors, supporting synaptic plasticity, and modulating brain blood flow (Bustamante-Barrientos et al., 2021). In support of this statements, a meta-analysis showed that a hormone replacement therapy in post-menopausal women protected against AD and PD (Song et al., 2020). Furthermore, epidemiological studies showed that PD for example is more prevalent in men than in women, which could be explained by the protective effect of estrogens in the brain (Bourque et al., 2019). Even before the COVID-19 pandemics it was already reported that ovariectomised or antioestrogen-treated female mice had more severe coronavirus infections than control mice (Channappanavar et al., 2017). Similarly, women taking oral contraceptives had reduced COVID-19 risk (Costeira et al., 2021). Furthermore, women with high levels of estradiol had a lower risk of developing severe symptoms and an even lower incidence of death due to COVID-19 (Ramírez-de-Arellano et al., 2021). Based on these findings repurposing the estrogen receptor modulator raloxifene was suggested as an option to treat COVID-19 (Allegretti et al., 2022). All things considered, aberrant estrogen signaling could contribute to neurodegeneration in COVID-19 patients.

“HIF-1 signaling pathway” was identified in the KEGG enrichment analysis. Hypoxia-inducible factor 1 (HIF-1) is a transcription factor functioning as a master regulator of oxygen homeostasis and plays a pivotal role in hypoxic environment. Multiple levels of evidence highlight the importance of HIF-1 in COVID-19 pathophysiology. Metabolic shift in immune cells of COVID-19 infected lungs is triggered by HIF-1α, which was observed on both transcriptomic and proteomic levels. This shift allows cells to survive in the hypoxic environment (Codo et al., 2020). Severe COVID-19 cases had higher expression of HIF-1 target genes (Appelberg et al., 2020). HIF-1α is constitutively expressed, but its expression can be increased upon PI3K/AKT and ERK activation as well (McGettrick and O'Neill, 2020). Another expression study correlated induced HIF-1α expression after COVID-19 infection with elevated inflammatory parameters, such as IL-6, interleukin 10, and TNFα (Tian et al., 2021). The role of HIF-1α in COVID-19 infection can be further elucidated with hypoxic induction of HIF-1α or with its pharmacological inhibition, showing that activation of oxygen-sensing pathways can hamper SARS-CoV-2 cell entry and viral replication (Wing et al., 2021). In neurodegenerative diseases, HIF-1 is well studied in AD, PD and ALS. HIF-1 induction in AD mouse reduces clustering of amyloid associated microglia and correlates with amyloid neuropathology in human hippocampus (March-Diaz et al., 2021). Although hypoxia has not been directly linked to PD, multiple shared genetic and metabolic features can be observed in HIF-1 signaling pathway and PD (Lestón Pinilla et al., 2021). In ALS mice, hypoxia has been associated with neuronal death, muscular and cognitive dysfunction (Kim et al., 2013). All of the above indicates that HIF-1 could present a shared pathway between COVID-19 and neurodegeneration and should be further explored.

“Neurotrophin signaling pathway” was identified in the KEGG pathway analysis as well. Neurotrophins are a family of proteins, involved in control of survival, development and function of neurons in both the peripheral and the CNS (Reichardt, 2006). Disrupted blood levels of β-nerve growth factor (β-NGF), an initial metabolite in neurotrophin signaling pathway, have been observed in mild SARS-CoV-2 patients (Usai et al., 2021). Besides NGF, brain-derived neurotrophic factor (BDNF) is the most studied neurotrophin in neurodegeneration. BDNF has been linked to microglial activation in mice (Wu et al., 2020). A causative *BDNF* rs6265 polymorphism is associated with cognitive decline and increased AD risk (Belbin et al., 2019; Lim et al., 2022). The same *BDNF* polymorphism was associated with PD and its symptoms (Cagni et al., 2017; Mercado et al., 2021), while precision medicine application was also suggested to personalize treatment of PD patients (Fischer et al., 2020). Furthermore, serum BDNF was increased in MS patients (Islas-Hernandez et al., 2018).

The enrichment of different neuroprotective pathways emphasizes the important role of mechanisms, involved in neuronal damage reduction. The interplay between neuroprotection and COVID-19 could also be further investigated in search for therapeutic applications.

#### 4.2.4. Viral infection pathways

The pathway of “Hepatitis B” has been enriched in the KEGG pathway analysis. The impaired liver function, although poorly understood, is an important clinically significant outcome after COVID-19 infection (da Mata et al., 2021; Yu et al., 2021). COVID-19 can also trigger hepatitis B reactivation, but no direct pathophysiological overlap between both diseases has been reported to date (Aldhaleei et al., 2020; Wu et al., 2021). Hepatitis B was also proposed as one of COVID-19 risk factors for severe disease outcome (Wang J. et al., 2021). Interestingly, a hepatitis B derived protein has been proposed as a promising therapeutic inhibitor of COVID-19 induced inflammation in lungs (Choi et al., 2021). Similarly, pathophysiological link between hepatitis B and neurodegenerative diseases remains unclear. Hepatitis B was proposed as increased risk factor for PD (Pakpoor et al., 2017), while increased risk was not observed in AD (Choi et al., 2021).

Another virus related pathway enriched in the KEGG analysis was the “HTLV-I infection” pathway. The human T-cell leukemia virus type I (HTLV-1) can cause several fatal diseases such as adult T-cell leukemia or HTLV-1 associated myelopathy/tropical spastic paraparesis. Although the majority of HTLV-1 carriers remain asymptomatic throughout their lives, they are at higher risk of acquiring different opportunistic infections (Olière et al., 2011). A case report of COVID-19 infected HTLV-1 carrier raises attention to potential long-term steroid use for secondary infection prevention (Enomoto et al., 2022). Although myelopathy can be a shared condition between HPTV-1 and MS, to date no solid evidence linking the diseases can be found.

Both viral infection pathways show significant enrichments in the pathway analysis. However, this could be due to the fact that similar pathways are activated upon viral infection and further studies will have to be conducted to establish their role in COVID-19 or neurodegeneration.

#### 4.2.5. Cellular senescence pathway

Cellular senescence was a significantly enriched pathway in the generated list of miRNA targets as well. Senescence is a phenomenon featuring discontinuation of cell divisions. Senescence is in its core a beneficial process, with the aim to get rid of the damaged cells by triggering the immune system and then replacing them. However, when this cell turnover system is inefficient, the senescent cells start accumulating, which contributes to the aging phenotype (López-Otín et al., 2013). Cells in senescent state accumulate in the aged tissue and particularly senescent astrocytes and microglia contribute to neurodegeneration. Some senescent cells can secrete cytokines and chemokines, which stimulate the neighboring cells to release ROS. Accumulation of such senescent cells increases the risk for developing a more severe COVID-19 and also a longer course of the disease with symptoms of neurodegeneration (Wissler Gerdes et al., 2022). A specific mechanism of cell senescence in COVID-19 has been described. As viruses replicate more efficiently in iron-rich senescent cells, they act towards changing the cell’s phenotype to make a more favourable environment for their replication. This predisposes the CNS to immune dysfunction and neurodegeneration (Sfera et al., 2021). All of the above indicates that cellular senescence is an important pathway that could contribute to neurodegenerative consequences of COVID-19.

## 5. Future perspectives

Since a significant amount of COVID-19 patients develop neurological symptoms, especially symptoms of neurodegenerative nature, it would be highly relevant to find biomarkers of neurodegenerative processes in COVID-19. The latter would enable identification of COVID-19 patients at increased risk of neurodegenerative diseases as a late COVID-19 consequence. Furthermore, elucidation of shared pathways between COVID-19 and neurodegeneration would open a window of opportunity for identification of novel drug targets within these pathways or drug repurposing to prevent or alleviate progression of COVID-19 to neurodegeneration.

Presented systematic search adopted a strategy of deploying miRNA molecules for identification of shared pathways between both pathologies and for identification of predictive biomarkers of neurodegeneration as a COVID-19 sequela. The procedure to obtain miRNAs and assess their expression in patient’s plasma or serum would be rather non-invasive, fast, and cost-effective. The three identified miRNAs (hsa-miR-34a, hsa-miR-132, and hsa-miR-155) could also present novel drug targets or novel drug compounds to alleviate neurodegeneration with acting on signaling cascades within shared pathways. In this study we only focused on the five most prevalent neurodegenerative diseases, although neurodegeneration due to COVID-19 could presumably also be presented in other forms, such as HIV encephalitis, which is a type of a virus-induced neurocognitive impairment.

Similar to our results, it has already been suggested that serum and plasma biomarkers could support detection of persistent neuroinflammation and neurodegeneration in COVID-19 patients (Sun et al., 2021; Chandra and Johri, 2022).Elevated cytokines are general markers of inflammatory process, which is driven by SARS-CoV-2, and they have been associated with higher chance of neurological sequelae development (Haidar et al., 2021; Sun et al., 2021). The exact mechanism on how SARS-CoV-2 induces changes in the brain, leading to potential neurodegeneration, is not fully elucidated. However, the three identified miRNAs shared between COVID-19 and neurodegeneration could be used as screening biomarkers to identify COVID-19 patients with increased risk for neurodegeneration. If the risk for neurodegeneration would be elevated based on the screening test, then more specific tests to decipher between different neurodegenerative pathologies could be applied afterwards. Specific markers of neurodegeneration in plasma could be measured, such as total tau (t-tau), phosphorylated tau-181 (p-tau_181_), glial fibrillary acidic protein (GFAP), neurofilament light chain (NfL), ubiquitin carboxyterminal hydrolase L1 (UCHL1), and Aβ (Aβ_40_, Aβ_42_) for dementia-related disorders (Frontera et al., 2022).

Together with dysregulated expression of individual miRNAs, the identification of neuroinflammation-related enriched pathways based on the shared miRNA targets between both pathologies further imply the importance of neuroinflammation in COVID-19. For more precise evaluation of their effect, future studies should also focus on distinct disease stages as well, since specific miRNAs can be regulated differently during disease progression. In the outline of our study, we focused on dysregulated miRNAs in neurodegenerative diseases in general. Stratification of pathologies to different stages of the disease could lead to more precise and targeted studies in the future. Additionally, miRNAs that remain dysregulated even after the recovery from COVID-19 infection could be of interest in potential follow-up studies in the same patient cohorts. Certain studies have already followed the miRNA expression longitudinally. Two studies did not report any miRNAs that remained differentially expressed even after the recovery (Zheng et al., 2020; Farr et al., 2021), while among others hsa-miR-155-5p remained upregulated in the post-acute phase of the disease in comparison to controls in one study (Donyavi et al., 2021).

Additionally, expression of several target genes of identified miRNAs has been shown to be dysregulated in COVID-19 patients on both mRNA and protein levels, which further supports our findings. The top identified miRNAs and pathways could thus serve as a basis to search for novel drug targets for prevention of neurodegeneration in COVID-19 or to screen for existing compounds targeting these pathways (Panda et al., 2022). The most promising enriched pathways, identified with our approach, could be the PI3K/AKT and MAPK signaling as neuroinflammation promoting pathways, and FOXO signaling as a neuroprotective pathway. Since host-derived miRNAs play essential role in limiting viral replication, they could potentially be utilized as drug compounds to normalize perturbations in signaling pathways due to the underlying pathology. Furthermore, compounds targeting the identified miRNAs and pathways could be studied as potential very specific anti-neuroinflammatory drugs, which consequently affect the development of neurodegeneration (Arghiani et al., 2021; Bautista-Becerril et al., 2021; Panda et al., 2022). Presumably, these drugs would be administered after recovery from COVID-19, in case the identified neurodegeneration-related biomarkers would still be elevated.

## 6. Conclusion

Deciphering the shared landscapes between COVID-19 and neurodegeneration could foster identification of COVID-19 patients with increased risk for AD, PD, ALS, MS, and HD development. It could also enable drug discovery or drug repurposing for prevention or delay of consequential neurodegeneration in COVID-19. Studying shared miRNA landscapes presents a promising approach to tackle this challenge as it serves us with potential specific neuronflammation-related markers of neurodegeneration. Further studies are needed to confirm these findings in a clinical setting of COVID-19 patients.

## Data availability statement

The original contributions presented in the study are included in the article/Supplementary material, further inquiries can be directed to the corresponding author.

## Author contributions

SR proposed the analysis workflow. SR, DV, and KG performed the literature, database research, and wrote the paper. DV and KG performed the database mining and the pathway enrichment analysis. SR designed the figures and the graphical abstract. VD supervised the writing, edited the final manuscript, and provided the funding. All authors contributed to the article and approved the submitted version.

## Funding

This work was supported by the Slovenian Research Agency (ARRS) (grant no. P1-0170).

## Conflict of interest

The authors declare that the research was conducted in the absence of any commercial or financial relationships that could be construed as a potential conflict of interest.

## Publisher’s note

All claims expressed in this article are solely those of the authors and do not necessarily represent those of their affiliated organizations, or those of the publisher, the editors and the reviewers. Any product that may be evaluated in this article, or claim that may be made by its manufacturer, is not guaranteed or endorsed by the publisher.

## Supplementary material

The Supplementary material for this article can be found online at: https://www.frontiersin.org/articles/10.3389/fnmol.2023.1123955/full#supplementary-material

## Figures and Tables

**Table:** 

Aβ	Amyloid-β
ACHE	Acetylcholinesterase
AD	Alzheimer’s disease
ADAM10	ADAM metallopeptidase domain 10
AKT	Protein kinase B
ALS	Amyotrophic lateral sclerosis
AQP1	Aquaporin 1
AP-1	Activator protein 1
APP	Amyloid precursor protein
AT1	Angiotensin II receptor type 1
BACE1	Beta-secretase 1
BCL2	BCL2 apoptosis regulator
BDNF	Brain-derived neurotrophic factor
β-NGF	β-Nerve growth factor
CNS	Central nervous system
DAVID	Database for Annotation, Visualization and Integrated Discovery
ERK1/2	Extracellular signal-regulated kinase 1 and 2
FDR	False discovery rate
FOXO	Class O of forkhead box transcription factors
GFAP	Glial fibrillary acidic protein
HD	Huntington’s disease
HIF-1	Hypoxia-inducible factor 1
HMDD	Human microRNA Disease Database
HO-1	Haem oxygenase 1
HTLV-1	Human T-cell leukemia virus type I
IL-1β	Interleukin 1β
IL-2	Interleukin 2
IL-4	Interleukin 4
IL-5	Interleukin 5
IL-6	Interleukin 6
IL6ST	IL-6 Signal transducer
IL-10	Interleukin 10
IL13RA1	Interleukin 13 receptor subunit alpha 1
IRF1	Interferon regulatory factor 1
JNK	c-Jun N-terminal kinase
KEAP1	Kelch-like ECH-associated protein 1
KEGG	Kyoto Encyclopedia of Genes and Genomes
MAPK1	Mitogen-activated protein kinase
MMP9	Matrix metallopeptidase 9
mRNA	Messenger RNA
MS	Multiple sclerosis
mTOR	Mammalian target of rapamycin
NF-κB	Nuclear factor kappa-light-chain-enhancer of activated B cells
NfL	Neurofilament light chain
NLRP3	NLR family pyrin domain containing 3
NOS1	Nitric oxide synthase 1
NRF2	Nuclear factor erythroid 2-related factor 2
PD	Parkinson’s disease
PS-1	Presenilin-1
PI3K	Phosphatidyl-inositol-3-kinase
PICALM	phosphatidylinositol binding clathrin assembly protein
p-tau_181_	Phosphorylated tau-181
PTEN	Phosphatase and tensin homolog
ROS	Reactive oxygen species
SIRT1	Sirtuin 1
SHC3	SHC-transforming protein 3
SHIP1	Inositol polyphosphate-5-phosphatase D
SOCS1	Suppressor of cytokine signaling 1
SORL1	Sortilin related receptor 1
TDP-43	TAR DNA-binding protein 43
TMPRSS2	Transmembrane protease, serine 2
TLR	Toll-like receptor
TNFα	Tumor necrosis factor
TREM2	Triggering receptor expressed on myeloid cells 2
t-tau	Total tau
UCHL1	Ubiquitin carboxyterminal hydrolase L1
